# Instance dataset for resource-constrained project scheduling with diverging material flows

**DOI:** 10.1016/j.dib.2023.109279

**Published:** 2023-05-29

**Authors:** Marco Gehring, Rebekka Volk, Frank Schultmann

**Affiliations:** Karlsruhe Institute of Technology, Institute for Industrial Production (IIP), Hertzstr. 16, 76187 Karlsruhe, Germany

**Keywords:** Storage facilities, Storage constraints, Cumulative resources, Dismantling projects, Benchmark instances

## Abstract

This data article describes an instance dataset motivated by the problem of scheduling a project with diverging material flows. Such material flows are released during the execution of the project and are subject to limited processing and storage capacities. Typical examples are nuclear dismantling or other deconstruction/demolition projects, where large amounts of material must be classified, scanned for hazardousness, and processed accordingly. The problem setting is mathematically described as a resource-constrained project scheduling problem with cumulative resources (RCPSP/c). The RCPSP/c deals with finding a project schedule with minimal makespan that satisfies temporal, renewable resource, and cumulative resource constraints. In total, the dataset comprises 192 artificially generated instances that are suitable for testing models and solution methods. In addition, we provide our best found solution for each instance and different modeling variants (e.g., for two types of objective functions). These solutions were computed by heuristic solution methods. The dataset serves as a benchmark for researchers evaluating the performance of solution methods for the RCPSP/c or the more general problem class with resources that can be produced and consumed.

**Specifications Table**

**Table:** 

Subject	Operations Research
Specific subject area	Resource-constrained project scheduling with cumulative resources
Type of data	1) Archive files (.zip) containing comma-separated values files (.csv) for instances and solutions 2) Excel file (.xlsx) providing an overview of instances and solutions 3) Archive file (.zip) containing Java source files (.java) and class files (.class) for a program to check the feasibility of solutions
How the data were acquired	Instances were artificially generated by a Java program. Solutions were computed by a Java program using different heuristic solution methods (cf. [1]). All programs were run on an AMD Ryzen 9 (4.0 gigahertz, 12 cores) with 128 gigabyte of RAM.
Data format	1) Raw 2) Analyzed 3) Java program
Description of data collection	We defined nine parameters for describing the characteristics of an instance. For each parameter, we defined a set of possible levels. For each combination of the parameter levels, we artificially generated a pair of instances. Each pair consists of an instance with granular and an instance with aggregated operations. In total, we got 96 pairs (i.e., 192 instances). And, we computed solutions for each instance and different modeling variants using heuristic solution methods.
Data source location	Institution: Karlsruhe Institute of Technology City/Town/Region: Karlsruhe Country: Germany
Data accessibility	Repository name: Mendeley Data Data identification number: 10.17632/z9gfh66mj4.1 Direct URL to data: https://doi.org/10.17632/z9gfh66mj4.1
Related research article	M. Gehring, R. Volk, F. Schultmann, On the integration of diverging material flows into resource-constrained project scheduling, Eur J Oper Res 303 (2022) 1071-1087. https://doi.org/10.1016/j.ejor.2022.03.047

## Value of the Data

• The dataset (cf. [2]) contains problem instances of the resource-constrained project scheduling problem with cumulative resources (RCPSP/c) presented by [1]. These instances can be used to evaluate the performance of suitable solution methods.
• Since the RCPSP/c is a particular case of a scheduling problem with resources that can be produced and consumed, the provided instances additionally serve as test instances for this general problem class, which has been introduced and discussed in several publications, such as [3], [4], [5], [6], [7], [8], [9].
• The best found solutions using heuristic methods are also provided and can be considered as a benchmark.
• The dataset includes a program for checking the feasibility of solutions. It helps researchers verifying the correct implementation of the problem formulation and the functionality of their solution methods.
• The dataset will benefit researchers involved in developing solution methods for the scheduling problem with resources that can be produced and consumed (also called ‘cumulative resources’, ‘reservoirs’, or ‘storage resources’).

## 1 Objective

The dataset has been generated to evaluate the performance of solution methods for the resource-constrained project scheduling problem with cumulative resources (RCPSP/c) presented by [1]. As a supplement to [1], this data article provides details about (i) the naming of instance and solution files concerning different modeling variants, (ii) the format of the instance and solution files, (iii) the instance generation procedure using an illustrative example, and (iv) the problem formulation concerning different modeling variants. The objective of this data article is to support researchers in accessing and reusing the generated dataset. For example, following the description of how to retrieve the parameters of the RCPSP/c from the provided CSV files, researchers can implement their own parser for reading the instance data.

## 2 Data Description

The dataset consists of three archive files and one Excel file. The archive file ‘Instances.zip’ contains instances of the *resource-constrained project scheduling problem with cumulative resources (RCPSP/c)* presented in [1]. The archive file ‘Solutions.zip’ contains the best found solutions for the instances in ‘Instances.zip’. The Excel file ‘Overview.xlsx’ provides an overview of instance characteristics and objective values of the best found solutions. The archive file 'SolutionCheck.zip' contains a Java program's source code for checking the solutions' feasibility. The dataset can be downloaded from [2].

The RCPSP/c is an extension of the well-known resource-constrained project scheduling problem (RCPSP) (cf. [10]). It aims to include diverging material flows, typically occurring in large-scale dismantling projects (e.g., nuclear dismantling projects), into the RCPSP. These material flows can impose delays on the project schedule due to limited processing and storage capacities. More formally, the RCPSP/c simultaneously deals with

(i) scheduling a project using a set of (project) activities and
(ii) scheduling the processing of material flows using a set of operations.

The conceptual problem formulation is provided in Appendix A.

The RCPSP/c is computationally challenging due to the cumulative resource type required for modeling the limited storage capacities. In contrast to the renewable resource type considered in the RCPSP, the availability of cumulative resources depends on all previous requirements. Due to its application-oriented formulation, the RCPSP/c can be considered a particular case of the general class of scheduling problems with resources that can be produced and consumed. Thus, the presented dataset also serves as a benchmark dataset for this general problem class, which has been introduced and discussed in several publications, such as [3], [4], [5], [6], [7], [8], [9]. However, until now, no consistent convention has been established for the verbal and formal formulation of such problems and, to the best of our knowledge, no general test instances are publicly accessible so far. For example, [3], [4], [5] use the term ‘cumulative resource’, [6] refers to ‘reservoirs’, [7,8] speak of ‘consumption and production of resources’, and [9] refers to ‘storage resources’.

For modeling the processing of material flows, the term ‘operation’ has been introduced in [1]. However, there are different modeling variants depending on the **type of operation** (cf. [1], Section 7): Each processing step of each material unit can be modeled as a single operation. This type is called *granular operations* (short: ‘gra’). Alternatively, processing several material units can be modeled together as one operation, in which case we speak of *aggregated operations* (short: ‘agg’). The type of operation has an impact on the problem formulation, as can be seen in Appendix A.

Each instance is recorded as a separate file within ‘Instance.zip’. Instances differ depending on the type of operation. Thus, instance files are named with ‘[number]_{gra, agg}.csv’, where [number] ranges from 1 to 96. In total, the ‘Instances.zip’ archive file contains 192 instance files. Since instances with aggregated operations are derived from instances with granular operations (cf. Section 3), each instance pair represents the same problem setting. For example, instances ‘1_gra.csv’ and ‘1_agg.csv’ represent a project with 30 activities, 350 released material units, the same processing and storage capacities, and so on. They only differ in the way operations were generated. On average, the number of operations in an instance with aggregated operations is 93% lower than in the corresponding instance with granular operations.

The Excel file ‘Overview.xlsx’ provides all details about the instances. Each line in this file refers to one of the 96 instance pairs. Column B indicates the PSPLIB-instance (cf. [11]), which has been used as the starting point for generating the instance pair in this dataset. Columns C to K indicate the levels of the parameters used for the instance generation (cf. [1], Section 8.1). Columns L to N provide additional characteristics of the generated instances.

The comma-separated value (CSV) format has been chosen for recording the instance files, with the semicolon serving as the delimiter. Table 1 describes the structure of each instance file. The first column in Table 1 indicates the number of consecutive lines to be traversed so that problem parameters listed in the second and third column can be retrieved. Each file also contains header lines, as indicated in Table 1. These headers are for comprehension purposes only, i.e., they can be skipped by a parser.

**Table 1:** Structure of the instance files.

	Problem parameters that can be retrieved from these lines	
Number of consecutive lines	Description	Corresponding notation (cf. Appendix A and [1])
1	Number of activities (including fictitious start and end activity)	i=0,…,I+1
1	Number of operations (including fictitious end operation)	$j=o_{1},\;\ldots ,\;o_{J+1}$
1	Number of renewable resources	$R^{\alpha }$
1	Number of cumulative resources	$R^{\gamma }$
2	[Headers]	
I+2	Activity name, activity duration, number of successors in precedence relations, list of successors in precedence relations	$d_{i}$, E
2	[Headers]	
J+1	Operation name, duration, number of successors in flow-induced precedence relations, list of pairs of successor and minimum time lag in flow-induced precedence relations	$d_{j},\;E^{flow},\;d_{jj}^{min\;}$
2	[Headers]	
I+2	Activity name, number of successors in release relations, list of pairs of successor and minimum time lag in release relations	$E^{rel},\;d_{ij}^{min\;}$
2	[Headers]	

I+2	Activity name, renewable resource requirement for each renewable resource, cumulative resource requirement for each cumulative resource	$r_{ik}^{\alpha },\;f_{ik}\;$
2	[Headers]	
J+1	Operation name, renewable resource requirement for each renewable resource, cumulative resource requirement for each cumulative resource	$r_{jk}^{\alpha },\;r_{jk}^{\gamma }$
2	[Headers]	
1	Maximum availability for each renewable resource, maximum inventory for each cumulative resource	$R_{k}^{\alpha },R_{k}^{\gamma }$

For positive cumulative resource requirements, an activity or operation ‘replenishes’ material units into a cumulative resource. For negative cumulative resource requirements, an operation ‘depletes’ material units from a cumulative resource. Activities may not deplete by definition since we deal with diverging material flows.

We use parameter $f_{ik}$ here instead of $f_{iw}$ in [1], where w is the index of a material flow path in the set of material flow paths W. The parameters $f_{ik}$ result from converting the data structure used in [1] by setting $f_{ik}:=\sum_{w\in W|k_{1}\left(w\right)=k}f_{iw}$ for all activities i=0,…,I+1 and all cumulative resources $k\in R^{\gamma }$, where $k_{1}\left(w\right)$ denotes the first cumulative resource in material flow path w∈W. That is, we sum over all material units following a material flow path starting with k. This conversion helps us to simplify the problem formulation (cf. Appendix A) and the structure of the instances since we no longer have to deal with material flow paths.

There are different modeling variants for cumulative resources depending on the **type of work progress** involved in the problem setting (cf. [1], Section 7): Work can either progress in a *stepwise* (short: ‘step’) fashion, which results in cumulative resource requirements occurring at the start and end times of activities or operations. Such models were investigated by [3,5,7], for example. Alternatively, work can progress *linearly* (short: ‘lin’) with time, which results in a uniform distribution of the cumulative resource requirements over the execution time of activities or operations. This modeling variant has been introduced by [4]. The type of work progress has an impact on the problem formulation, as can be seen in Appendix A.

In its original formulation in [1], the objective of the RCPSP/c is to minimize the *project makespan* (short: ‘project’). Since the project only consists of activities, the project makespan equals the latest end time of all activities. An alternative **type of objective function** is to minimize the *total makespan* (short: ‘total’), which equals the latest end time of all activities and operations (cf. [1], Section 8.4.4).

Each best found solution is recorded as a separate file within ‘Solutions.zip’. Solutions differ depending on the type of operation, the type of work progress, and the type of objective function. Thus, solution files are named with ‘[number]_{gra, agg}_{lin, step}_{project, total}_solution.csv’, where [number] ranges from 1 to 96. In total, the ‘Solutions.zip’ archive file contains 768 solution files.

The CSV format has been chosen for recording the solution files, with the semicolon serving as the delimiter. Analogous to Table 1, Table 2 describes the structure of each solution file.

**Table 2:** Structure of the solution files.

	Decision variables that can be retrieved from these lines	
Number of consecutive lines	Description	Corresponding notation (cf. Appendix A and [1])
1	Objective value	z or $z^{\prime }$ (depending on the chosen objective function)
1	Project makespan (excluding operations)	$S_{I+1}$
1	Total makespan (including operations)	$\max\{S_{I+1},S_{o_{J+1}}^{o}\}$
2	[Headers]	
I+2	Activity name, start time, end time	$S_{i}$
2	[Headers]	
J+1	Operation name, start time, end time	$S_{j}^{o}$

The Excel file ‘Overview.xlsx’ lists the objective values of all best found solutions in columns O to W. Besides, it provides two lower bounds $LB_{\text{PSPLIB}}$ and $LB_{flow}$ (cf. [1], Section 8.3) in columns X to AB. These bounds differ depending on the type of objective function.

To check whether a solution is feasible for a specific instance, we provide a Java program named ‘SolutionCheck’ as a part of the dataset. It enables researchers to check the feasibility of their self-computed solutions. This helps them verifying the correct implementation of the problem formulation and the functionality of their solution methods.

The Java program was written with the Eclipse IDE and the Eclipse project was exported as an archive file ‘SolutionCheck.zip’. It can be reimported into Eclipse by right-clicking within the package explorer and selecting ‘Existing projects into Workspace.’ Other IDEs offer similar wizards for importing source code.

The program consists of four classes split into three packages. No graphical user interface is provided. All settings must be specified in the source code of the class ‘MainClass.java’ in package ‘main’. Here, the pathname of the instance and the solution to be checked must be entered. Furthermore, the type of operation (‘gra’, ‘agg’) and the type of work progress (‘lin’, ‘step’) must be chosen correctly. Inline comments explain all necessary adaptions in the source code. After running the program, it prints ‘Solution is feasible’, or information about the first violated constraint to the console. Please note that the instance and solution files must match each other. The solution file must be recorded according to the same structure as the files provided in ‘Solutions.zip’ (cf. Table 2). Otherwise, exceptions might occur.

## 3 Experimental Design, Materials and Methods

The procedure for generating instances with granular operations is described in [1], Section 8.1. As explained there, we employ two parameter groups for characterizing the instances: Project and material flow parameters. Table 3 summarizes these parameters along with the levels we used for the instance generation. We get $2^{5}\cdot 3=96$ combinations of all levels according to a full factorial experimental design. For each combination, we generated one instance, which is why our instance dataset comprises 96 instances.

**Table 3:** Parameters used for instance generation.

Parameter	Denotation	Levels
Project parameters		
$I\in Z_{>0}$	Number of activities	{30,120}
NC>0	Network complexity	{2.1}
RS∈[0,1]	Renewable resource strength	{0.5}
RFA∈[0,1]	Renewable resource factor	{0.5}
Material flow parameters		
$INV\in Z_{\geq 0}$	Maximum inventory of each cumulative resource	{200,1000}
$NREL\in Z_{\geq 0}$	Number of released material units by non-fictitious activities	{50,200}
PREL∈[0,1]	Portion of non-fictitious activities releasing material units	{0.25,1}
RFP∈[0,1]	Renewable resource factor for processing steps	{0,0.5}
$DUR\in R_{\geq 0}^{\left|P\right|}$	Duration vector for processing steps	$\left\{dur_{1},\;dur_{2},\;dur_{3}\right\}$

Since we use PSPLIB-instances as the starting point for generating our instances, the possible levels of project parameter I are restricted to the levels prescribed by the PSPLIB (i.e., {30,60,90,120}; cf. [11]). Of these, we chose the smallest and the largest value. We fixed project parameters NC, RS, and RFA to one single level since they do not considerably impact the performance of solution methods (cf. [1], Appendix A2).

For each material flow parameter, we limited ourselves to two or three levels. The reason for this is that with the given modeling variants (type of operation, type of work progress, and type of objective function) and with different solution methods, the number of required solving runs is a multiple of the number of instances. We chose 200 as the largest value for NREL, so that with PREL=1 and I=120, a total of 24,000 material units are released. For comparison: for the dismantling of reactor 2 of the Philippsburg nuclear power plant in Germany, it is stated that 15,590 tons of radioactive residues are released [12]. If we assume that one ton is modeled as one material unit, our largest instance with 24,000 material units is comparable to a large nuclear dismantling project. Another value of 50 exists for NREL, which allows for generating smaller instances. We chose 200 as the smallest value for INV, which means that with NREL=200, just the material released by one activity fits into a storage facility. With INV=1000, more storage capacity is available. For PREL, we selected the values 0.25 and 1, thus creating instances in which only some of the activities release materials, as well as instances in which all activities release materials. For RFP, we chose the values 0 and 0.5. In the case of RFP=0, there do not exist renewable resources used by both activities and operations. With RFP=0.5, on the other hand, we get instances in which activities and operations compete against renewable resources.

The set of processing steps is P={P1,…,P8}. It only serves as a pattern for generating operations as described in [1], Section 8.1, and is not part of the instance data. The levels for DUR are the vectors $dur_{1}=\left(0.06,\;0.06,\;\ldots ,\;0.06\right)$, $\mathrm{du}r_{2}=\left(0.1,\;0.08,\;0.06,\;0.1,\;0.04,\;0.1,\;0.02,\;0.02\right)$, and $\mathrm{du}r_{3}=\left(0.02,\;0.04,\;0.06,\;0.02,\;0.08,\;0.04,\;0.1,\;0.08\right).$ With $dur_{1}$, all processing steps require the same time. Vector $dur_{2}$ describes a situation where a processing bottleneck is in the upstream part of the material flows. With $dur_{3}$, a processing bottleneck is in the downstream part, respectively (cf. [1]).

We can graphically represent an instance of the RCPSP/c as a precedence network with node set $\left\{0,\ldots ,I+1\right\}\cup \left\{o_{1},\ldots ,o_{J+1}\right\}$ and arc set $E\cup E^{flow}\cup E^{rel}$. Precedence relations E may arbitrarily link activities if there is a single source node (= fictitious start activity 0) and a single sink node (= fictitious end activity I+1). However, no circles of precedence relations are allowed since this would cause infeasibility.

**Fig. 1:**
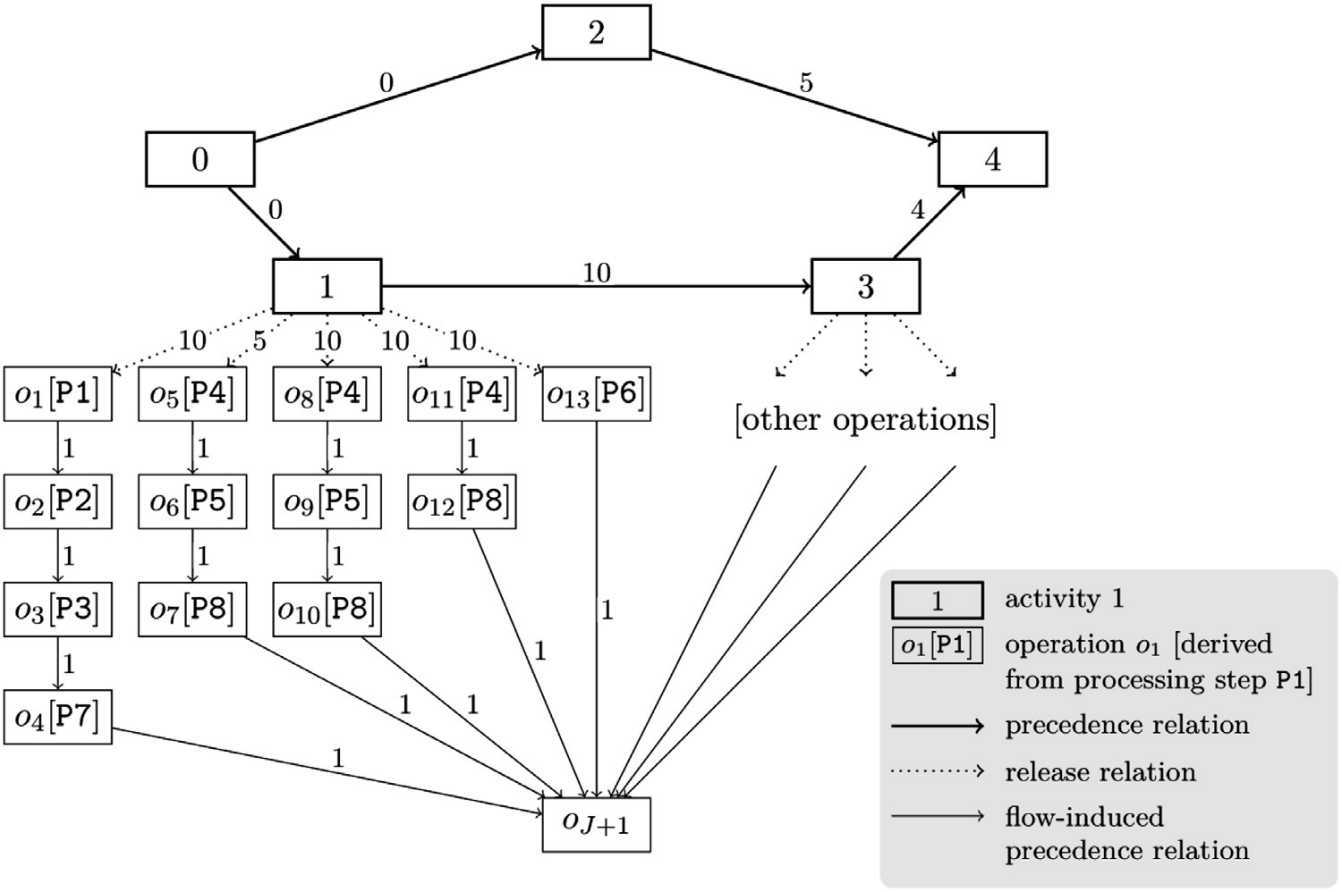
Exemplary precedence network of an instance with granular operations. This figure was published in [1], Copyright Elsevier.

Using the instance generation procedure defined in [1], flow-induced precedence relations $E^{flow}$ and release relations $E^{rel}$ form out-trees in the precedence network for each activity releasing material flows. In the case of granular operations, these out-trees only branch at their root, i.e., at the activity releasing the material flows. Fig. 1 (also shown in [1]) exemplarily depicts a precedence network of an instance with granular operations. Arc weights equal the minimum time lags or, in the case of precedence relations, the durations of the predecessors.

Instances with aggregated operations are derived from instances with granular operations: We replace several granular operations following the same upstream material flow path and modeling the same processing step by one aggregated operation. This aggregated operation spans the total duration and cumulative resource requirement of the replaced granular operations (cf. [1], Section 7). Renewable resource requirements remain unchanged.

**Fig. 2:**
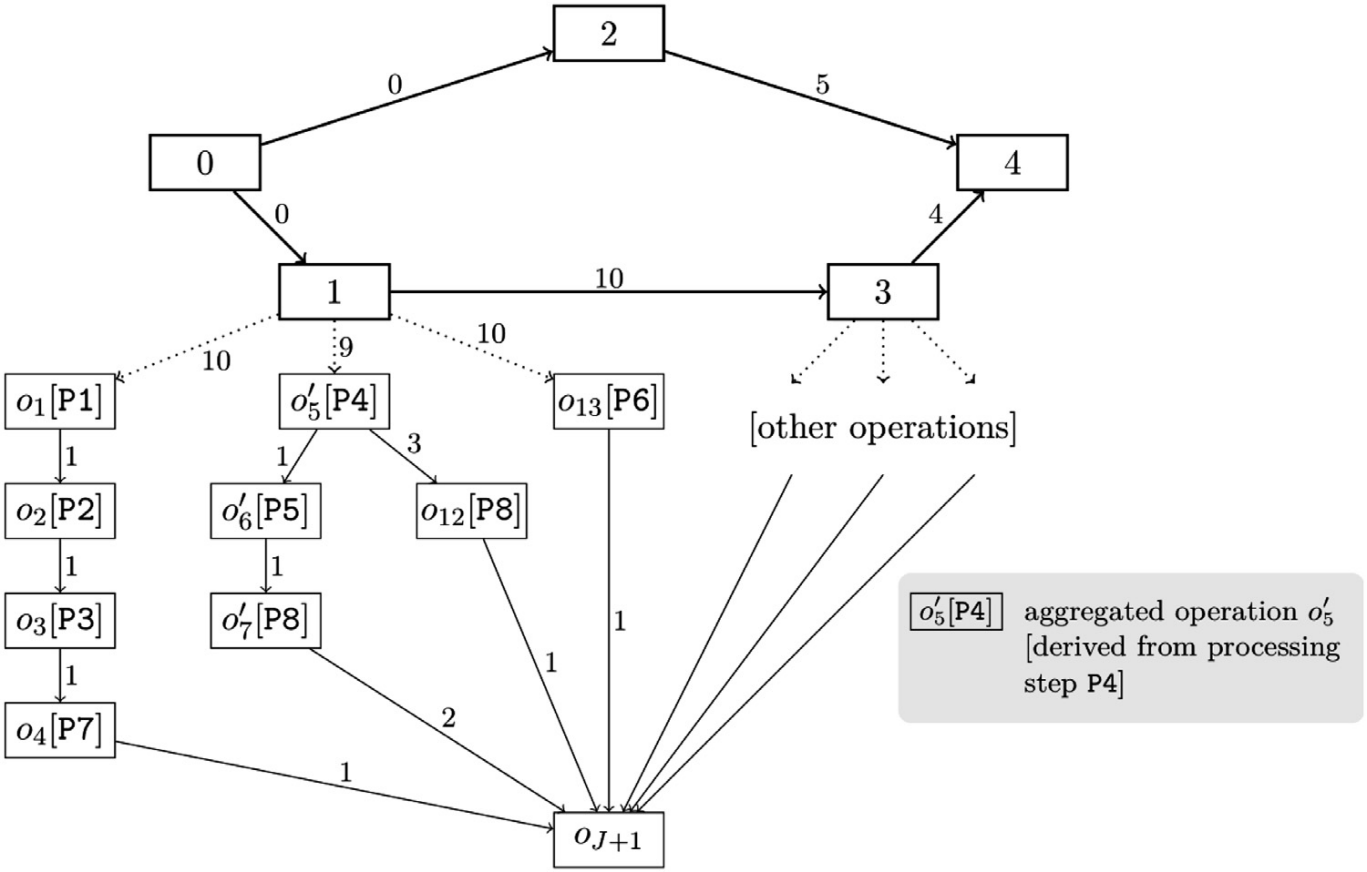
Exemplary precedence network of an instance with aggregated operations. This figure was published in [1], Copyright Elsevier.

Fig. 2 (also shown in [1]) depicts the precedence network of the instance with aggregated operations, which has been derived from the instance with granular operations in Fig. 1. For example, let us consider the three granular operations $o_{5}$, $o_{8}$, and $o_{11}$ in Fig. 1. Each of these granular operations models the processing of one material unit in processing step P4. Each of these granular operations is a direct successor of activity 1, i.e., follows the same upstream material flow path. The durations are $d_{o_{5}}=d_{o_{8}}=d_{o_{11}}:=1$. Let us further assume that each of these granular operations depletes one material unit from a cumulative resource S4 (i.e., $r_{j,S4}^{\gamma }=-1$ for $j=o_{5},o_{8},o_{11}$) and replenishes this material unit into another cumulative resource S5 (i.e., $r_{j,S5}^{\gamma }=1$ for $j=o_{5},o_{8},o_{11}$). And, each of these granular operations requires one unit of a renewable resource MA1 (i.e., $r_{j,\text{MA}1}^{\alpha }=1$ for $j=o_{5},o_{8},o_{11}$; MA = machine). Then, we can replace these three granular operations with one aggregated operation $o_{5}^{\prime }$, as shown in Fig. 2. This aggregated operation depletes three material units from S4 (i.e., $r_{o_{5}^{\prime },S4}^{\gamma }=-3$), replenishes three material units into S5 (i.e., $r_{o_{5}^{\prime },S5}^{\gamma }=3$), requires one unit of MA1 (i.e., $r_{o_{5}^{\prime },\text{MA}1}^{\alpha }=1$), and takes $d_{o_{5}^{\prime }}=3$ periods. Note that the resource requirements are not included in the figures.

**Fig. 3:**
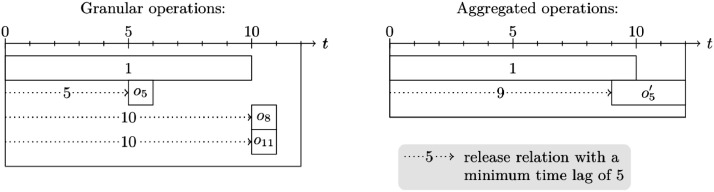
Exemplary illustration of minimum time lags for granular and aggregated operations.

When replacing granular operations with an aggregated operation, we set the minimum time lag between the predecessor and the aggregated operation in such a way that if we split the aggregated operations back into granular operations, all of the original time lags would be satisfied. Continuing the example from above, Fig. 1 indicates that $d_{1,o_{5}}^{min}=5$, $d_{1,o_{8}}^{min}=10$, and $d_{1,o_{11}}^{min}=10$. Then, we must set $d_{1,o_{5}^{\prime }}^{min}=9$ for the aggregated operation $o_{5}^{\prime }$ as the Gantt charts in Fig. 3 illustrate. For $d_{1,o_{5}^{\prime }}^{min}<9$, the minimum time lag $d_{1,o_{8}}^{min}=10$ would be violated if we split $o_{5}^{\prime }$ back into its underlying granular operations $o_{5}$, $o_{8}$, and $o_{11}$.

Using the formulations provided in Appendix A, all other constraints are also always satisfied if, for a given solution, we split aggregated operations back into their underlying granular operations. Hence, the optimal objective value of the problem with granular operations constitutes a lower bound for the objective value of the respective problem with aggregated operations (if the type of work progress and the type of objective function remain unchanged). For example, the objective value of an optimal solution for instance ‘1_gra.csv’ is a lower bound for the objective value of any solution for instance ‘1_agg.csv’.

For computing the best known solutions provided in ‘Solutions.zip’, we employed the following solution methods:

For instances with granular operations:

• The problem-specific schedule generation scheme (SGS) presented in [1], Section 6.
• A generic SGS following the idea of [13] (cf. [1], Appendix A6).

For instances with aggregated operations:

• An adaption of the problem-specific SGS presented in [1], Section 6, to instances with aggregated operations.
• A variant of the problem-specific SGS presented in [1], Section 6, where activities and operations are scheduled in an integrated way. That is, the decomposition into the two procedures SuperSchedule and SubSchedule in [1] has been removed. Instead, activities and operations are scheduled equally in SuperSchedule. We only implemented this variant for the case of aggregated operations because the number of operations is significantly smaller here than in the case of granular operations.
• A generic SGS following the idea of [13] (cf. [1], Appendix A6).

We implemented all these solution methods in Java without interfacing with external libraries. For each instance and each type of operation, type of work progress, and type of objective function, we ran each suitable solution method using a multi-start metaheuristic as described in [1], Section 8.2, with a time limit of ten minutes. We ran all computations on an AMD Ryzen 9 (4.0 gigahertz, 12 cores) with 128 gigabyte of RAM.

## Ethics Statements

The authors declare that their work complies with the ethical requirements for publication in Data in Brief. They confirm that their work does not involve human subjects, animal experiments, or any data collected from social media platforms.

## CRediT Author Statement

**Marco Gehring:** Conceptualization, Methodology, Software, Validation, Formal analysis, Investigation, Data curation, Writing – Original Draft, Writing – Review & Editing, Visualization. **Rebekka Volk:** Writing – Review & Editing, Supervision, Project administration, Funding acquisition. **Frank Schultmann:** Writing – Review & Editing, Supervision, Funding acquisition.

## Declaration of Competing Interest

The authors declare that they have no known competing financial interests or personal relationships that could have appeared to influence the work reported in this paper.

## Figures and Tables

**Table 4:** Notation of problem parameters.

Notation	Denotation
i=0,…,I+1	(project) activities; 0 and I+1 are the fictitious start and end activities with both durations and resource requirements of zero
$j=o_{1},\ldots ,o_{J+1}$	operations; $o_{J+1}$ is the fictitious end operation with both a duration and resource requirements of zero
$d_{i}\in Z_{\geq 0}$	duration of activity i
$d_{j}\in Z_{\geq 0}$	duration of operation j
$E\subset {\left\{0,\ldots ,I+1\right\}}^{2}$	set of precedence relations
$(i,i^{\prime })\in E$	precedence relation between activities i (predecessor) and $i^{\prime }$ (successor)
$E^{flow}\subset {\left\{o_{1},\ldots ,o_{J+1}\right\}}^{2}$	set of flow-induced precedence relations
$\left(j,j^{\prime }\right)\in E^{flow}$	flow-induced precedence relation between operations j (predecessor) and $j^{\prime }$ (successor)
$d_{jj^{\prime }}^{min}\in Z_{\geq 0}$	minimum time lag between the start times of operation j and operation $j^{\prime }$ in flow-induced precedence relation $\left(j,j^{\prime }\right)\in E^{flow}$
$E^{rel}\subseteq \left\{0,\ldots ,I+1\right\}\times \left\{o_{1},\ldots ,o_{J+1}\right\}$	set of release relations
$\left(i,j\right)\in E^{rel}$	release relation between activity i (predecessor) and operation j (successor)
$d_{ij}^{min}\in Z_{\geq 0}$	minimum time lag between the start times of activity i and operation j in release relation $\left(i,j\right)\in E^{rel}$
$R^{\alpha }$	set of renewable resources
$R_{k}^{\alpha }\in Z_{\geq 0}\;$	maximum availability of renewable resource $k\in R^{\alpha }$
$r_{ik}^{\alpha }\in Z_{\geq 0}$	number of units of renewable resource $k\in R^{\alpha }$ required by activity i (= renewable resource requirement)
$r_{jk}^{\alpha }\in Z_{\geq 0}$	number of units of renewable resource $k\in R^{\alpha }$ required by operation j (= renewable resource requirement)
$R^{\gamma }$	set of cumulative resources
$k\in R^{\gamma }$	cumulative resource
$R_{k}^{\gamma }\in Z_{\geq 0}\;$	maximum inventory of cumulative resource $k\in R^{\gamma }$
$f_{ik}\in Z_{\geq 0}$	number of units of cumulative resource $k\in R^{\gamma }$ required by activity i (= cumulative resource requirement)

$r_{jk}^{\gamma }\in Z$	number of units of cumulative resource $k\in R^{\gamma }$ required by operation j (= cumulative resource requirement)

**Table 5:** Notation of decision variables.

Notation	Denotation
$S_{i}$	start time of activity i
$S={\left(S_{i}\right)}_{i=0,\ldots ,I+1}$	schedule of activities
$S_{j}^{o}$	start time of operation j
$S^{o}={\left(S_{j}^{o}\right)}_{j=o_{1},\ldots ,o_{J+1}}$	schedule of operations

**Table 6:** Definitions of decision-dependent functions.

Function and definition	Denotation
$A^{\alpha }\left(S,t\right):=\left\{i\in \left\{0,\cdots ,I+1\right\}\;|\;S_{i}\leq t<S_{i}+d_{i}\right\}$	set of activities requiring renewable resources at time t, given schedule S
$A^{\alpha }\left(S^{o},t\right):=\left\{j\in \left\{o_{1},\cdots ,o_{J+1}\right\}\;|\;S_{j}^{o}\leq t<S_{j}^{o}+d_{j}\right\}$	set of operations requiring renewable resources at time t, given schedule $S^{o}$
$r_{k}^{\alpha }\left(S,t\right):=\sum_{i\in A^{\alpha }\left(S,t\right)}r_{ik}^{\alpha }$	total requirement of renewable resource $k\in R^{\alpha }$ by activities at time t, given schedule S
$r_{k}^{\alpha }\left(S^{o},t\right):=\sum_{j\in A^{\alpha }\left(S^{o},t\right)}r_{jk}^{\alpha }$	total requirement of renewable resource $k\in R^{\alpha }$ by operations at time t, given schedule $S^{o}$
$A^{\gamma }\left(S,t\right):=\left\{i\in \left\{0,\cdots ,I+1\right\}\;|\;0\leq S_{i}\leq t\right\}$	set of activities requiring cumulative resources at time t, given schedule S
$x_{i}\left(S,t\right):=\left\{ \begin{array}{cc} 0 & \text{if}\;t<S_{i},\\ 1 & \text{if}\;t\geq S_{i}+d_{i},\\ \left(t-S_{i}\right)/d_{i} & \text{otherwise} \end{array}\right.$	
	portion of activity i that has been completed at time t, given schedule S
$x_{j}\left(S^{o},t\right):=\left\{ \begin{array}{cc} 0 & \text{if}\;t<S_{j}^{o},\\ 1 & \text{if}\;t\geq S_{j}^{o}+d_{j},\\ \left(t-S_{j}^{o}\right)/d_{j} & \text{otherwise} \end{array}\right.$	
	portion of operation j that has been completed at time t, given schedule $S^{o}$

**Table 7:** Definitions of decision-dependent functions in the case of granular operations and linear work progress.

Function and definition	Denotation
$f_{k}^{\gamma }\left(S,t\right):=\sum_{i\in A^{\gamma }\left(S,t\right)}\left\lfloor f_{ik}\;x_{i}\left(S,t\right)\right\rfloor$	total requirement of cumulative resource $k\in R^{\gamma }$ by activities at time t, given schedule S
$A_{k}^{\gamma +}\left(S^{o},t\right):=\left\{j\in \left\{o_{1},\cdots ,o_{J+1}\right\}\;|\;r_{\mathrm{jk}}^{\gamma }=1\wedge 0\leq S_{j}^{o}+d_{j}\leq t\right\}$	set of replenishing operations requiring cumulative resources at time t, given schedule $S^{o}$
$A_{k}^{\gamma -}\left(S^{o},t\right):=\left\{j\in \left\{o_{1},\cdots ,o_{J+1}\right\}\;|\;r_{\mathrm{jk}}^{\gamma }=-1\wedge 0\leq S_{j}^{o}\leq t\right\}$	set of depleting operations requiring cumulative resources at time t, given schedule $S^{o}$
$r_{k}^{\gamma }\left(S^{o},t\right):=\sum_{j\in A_{k}^{\gamma +}\left(S^{o},t\right)}r_{jk}^{\gamma }+\sum_{j\in A_{k}^{\gamma -}\left(S^{o},t\right)}r_{jk}^{\gamma }$	total requirement of cumulative resource $k\in R^{\gamma }$ by operations at time t, given schedule $S^{o}$

**Table 8:** Definitions of decision-dependent functions in the case of granular operations and stepwise work progress.

Function and definition	Denotation
$f_{k}^{\gamma }\left(S,t\right):=\sum_{i\in A^{\gamma }\left(S,t\right)}f_{ik}$	total requirement of cumulative resource $k\in R^{\gamma }$ by activities at time t, given schedule S
$A_{k}^{\gamma +}\left(S^{o},t\right):=\left\{j\in \left\{o_{1},\cdots ,o_{J+1}\right\}\;|\;r_{\mathrm{jk}}^{\gamma }=1\wedge 0\leq S_{j}^{o}+d_{j}\leq t\right\}$	set of replenishing operations requiring cumulative resources at time t, given schedule $S^{o}$
$A_{k}^{\gamma -}\left(S^{o},t\right):=\left\{j\in \left\{o_{1},\cdots ,o_{J+1}\right\}\;|\;r_{\mathrm{jk}}^{\gamma }=-1\wedge 0\leq S_{j}^{o}\leq t\right\}$	set of depleting operations requiring cumulative resources at time t, given schedule $S^{o}$
$r_{k}^{\gamma }\left(S^{o},t\right):=\sum_{j\in A_{k}^{\gamma +}\left(S^{o},t\right)}r_{jk}^{\gamma }+\sum_{j\in A_{k}^{\gamma -}\left(S^{o},t\right)}r_{jk}^{\gamma }$	total requirement of cumulative resource $k\in R^{\gamma }$ by operations at time t, given schedule $S^{o}$

**Table 9:** Definitions of decision-dependent functions in the case of aggregated operations and linear work progress.

Function and definition	Denotation
$f_{k}^{\gamma }\left(S,t\right):=\sum_{i\in A^{\gamma }\left(S,t\right)}\left\lfloor f_{ik}\;x_{i}\left(S,t\right)\right\rfloor$	total requirement of cumulative resource $k\in R^{\gamma }$ by activities at time t, given schedule S
$A_{k}^{\gamma +}\left(S^{o},t\right):=\left\{j\in \left\{o_{1},\cdots ,o_{J+1}\right\}\;|\;r_{\mathrm{jk}}^{\gamma }>0\wedge 0\leq S_{j}^{o}\leq t\right\}$	set of replenishing operations requiring cumulative resources at time t, given schedule $S^{o}$
$A_{k}^{\gamma -}\left(S^{o},t\right):\left\{j\in \left\{o_{1},\cdots ,o_{J+1}\right\}\;|\;r_{\mathrm{jk}}^{\gamma }<0\wedge 0\leq S_{j}^{o}\leq t\right\}$	set of depleting operations requiring cumulative resources at time t, given schedule $S^{o}$
$r_{k}^{\gamma }\left(S^{o},t\right):=\sum_{j\in A_{k}^{\gamma +}\left(S^{o},t\right)}\left\lfloor r_{jk}^{\gamma }\;x_{j}\left(S^{o},t\right)\right\rfloor +\sum_{j\in A_{k}^{\gamma -}\left(S^{o},t\right)}\left\lceil r_{jk}^{\gamma }\;x_{j}\left(S^{o},t\right)\right\rceil$	total requirement of cumulative resource $k\in R^{\gamma }$ by operations at time t, given schedule $S^{o}$

**Table 10:** Definitions of decision-dependent functions in the case of aggregated operations and stepwise work progress.

Function and definition	Denotation
$f_{k}^{\gamma }\left(S,t\right):=\sum_{i\in A^{\gamma }\left(S,t\right)}f_{ik}$	total requirement of cumulative resource $k\in R^{\gamma }$ by activities at time t, given schedule S
$A_{k}^{\gamma +}\left(S^{o},t\right):=\left\{j\in \left\{o_{1},\cdots ,o_{J+1}\right\}\;|\;r_{\mathrm{jk}}^{\gamma }>0\wedge 0\leq S_{j}^{o}\leq t\right\}$	set of replenishing operations requiring cumulative resources at time t, given schedule $S^{o}$
$A_{k}^{\gamma -}\left(S^{o},t\right):=\left\{j\in \left\{o_{1},\cdots ,o_{J+1}\right\}\;|\;r_{\mathrm{jk}}^{\gamma }<0\wedge 0\leq S_{j}^{o}+d_{j}\leq t\right\}$	set of depleting operations requiring cumulative resources at time t, given schedule $S^{o}$
$r_{k}^{\gamma }\left(S^{o},t\right):=\sum_{j\in A_{k}^{\gamma +}\left(S^{o},t\right)}r_{jk}^{\gamma }+\sum_{j\in A_{k}^{\gamma -}\left(S^{o},t\right)}r_{jk}^{\gamma }$	total requirement of cumulative resource $k\in R^{\gamma }$ by operations at time t, given schedule $S^{o}$

**Table:** 

$\min_{\left(S,S^{o}\right)}\;$	Λ		(1a)
subjectto	$S_{i^{\prime }}\geq S_{i}+d_{i}$	$(\forall (i,i^{\prime })\in E);$	(1b)
	$S_{j^{\prime }}^{o}\geq S_{j}^{o}+d_{jj^{\prime }}^{min}$	$(\forall \left(j,j^{\prime }\right)\in E^{flow});$	(1c)
	$S_{j}^{o}\geq S_{i}+d_{ij}^{min}$	$(\forall \left(i,j\right)\in E^{rel});$	(1d)
	$r_{k}^{\alpha }\left(S,t\right)+r_{k}^{\alpha }\left(S^{o},t\right)\leq R_{k}^{\alpha }$	$(\forall k\in R^{\alpha },t\geq 0);$	(1e)
	$f_{k}^{\gamma }\left(S,t\right)+r_{k}^{\gamma }\left(S^{o},t\right)\leq R_{k}^{\gamma }$	$(\forall k\in R^{\gamma },t\geq 0);$	(1f)
	$S_{0}=0;$		(1g)
	$S_{i}\geq 0$	(i=1,…,I+1);	(1h)
	$S_{j}^{o}\geq 0$	$\left(j=o_{1},\ldots ,o_{J+1}\right)$	(1i)

## Data Availability

Instance dataset for resource-constrained project scheduling with diverging material flows (Original data) (Mendeley Data).

## Appendix A. Problem Formulation

In this appendix, the mathematical formulation of the underlying RCPSP/c is provided. Subsection A1 deals with the notation and definitions that apply to all modeling variants. Subsection A2 presents definitions that specifically depend on the chosen type of operation and type of work progress. Based on the two previous subsections, Subsection A3 provides the mathematical formulation of the RCPSP/c.

### A.1. Notation and general definitions

Table 4 presents the notation of problem parameters. Table 5 presents the notation of decision variables. Table 6 presents definitions of decision-dependent functions that apply to all modeling variants.

### A.2. Specific Definitions

#### A.2.1. Definitions in the case of granular operations and linear work progress

In addition to the general definitions in Table 6, Table 7 presents definitions of decision-dependent functions in the case of granular operations and linear work progress.

In the case of granular operations, each operation models the application of one processing step to one material unit. Consequently, $r_{jk}^{\gamma }\in \left\{-1,\;0,\;1\right\}$ holds for all operations j and all cumulative resources $k\in R^{\gamma }$. An operation depletes one material unit from a cumulative resource at its start, processes it, and replenishes this material unit into another cumulative resource at its end. This notion is reflected in the definitions of the functions$\;A_{k}^{\gamma +}\left(S^{o},t\right)$ and $A_{k}^{\gamma -}\left(S^{o},t\right)$ in Table 7. It is also possible that an operation depletes and replenishes from/into the same cumulative resource. For such an operation j, $r_{jk}^{\gamma }=0$ holds for all cumulative resources $k\in R^{\gamma }$ (cf. paragraph about ‘neutral operations’ in [1], Section 5).

Activities release material units uniformly distributed over their execution time. This notion is reflected in the definition of $f_{k}^{\gamma }\left(S,t\right)$ in Table 7, where the cumulative resource requirement $f_{ik}$ is multiplied with $x_{i}\left(S,t\right)$ (= the portion of activity i that has been completed at time t; see also [4]). Since we assume an indivisibility of the material units, the floor function ⌊·⌋ is included in this definition. It also simplifies the implementation because it prevents decimal numbers when dealing with the cumulative resource profile.

#### A.2.2. Definitions in the case of granular operations and stepwise work progress

In addition to the general definitions in Table 6, Table 8 presents definitions of decision-dependent functions in the case of granular operations and stepwise work progress.

Compared to the previous subsection, only the definition of $f_{k}^{\gamma }\left(S,t\right)$ differs: Due to the stepwise work progress, all activities release the material units at their start times. Therefore, the cumulative resource requirements $f_{ik}$ are fully counted into the sum expression (cf. [1], Section 7). Since $f_{k}^{\gamma }\left(S,t\right)$ in Table 7 is always lower than or equal to $f_{k}^{\gamma }\left(S,t\right)$ in Table 8, the optimal objective value of the problem with linear work progress constitutes a lower bound for the objective value of the respective problem with stepwise work progress.

#### A.2.3. Definitions in the case of aggregated operations and linear work progress

In addition to the general definitions in Table 6, Table 9 presents definitions of decision-dependent functions in the case of aggregated operations and linear work progress.

In the case of aggregated operations, each operation models the application of one processing step to an arbitrary number of material units. Consequently, $r_{jk}^{\gamma }\in Z$ holds for all operations j and all cumulative resources $k\in R^{\gamma }$. Similar to activities, these cumulative resource requirements are uniformly distributed over the execution times of operations. This is why the definition of $r_{k}^{\gamma }\left(S^{o},t\right)$ includes the factors $x_{j}\left(S^{o},t\right)$ in Table 9. For depletions ($r_{jk}^{\gamma }<0$), we use the ceil function ⌈·⌉ instead of the floor function. The definitions of the sets $A_{k}^{\gamma +}\left(S^{o},t\right)$ and $A_{k}^{\gamma -}\left(S^{o},t\right)$ also differ from the definitions given before. Both sets now include all operations that have started before a time t.

#### A.2.4. Definitions in the case of aggregated operations and stepwise work progress

In addition to the general definitions in Table 6, Table 10 presents definitions of decision-dependent functions in the case of aggregated operations and stepwise work progress.

If work progresses in a stepwise fashion, operations replenish cumulative resources at their start and deplete cumulative resources at their end. This notion is reflected in the definitions of the functions$\;A_{k}^{\gamma +}\left(S^{o},t\right)$ and $A_{k}^{\gamma -}\left(S^{o},t\right)$ in Table 10. Since $f_{k}^{\gamma }\left(S,t\right)$ and $r_{k}^{\gamma }\left(S^{o},t\right)$ in Table 9 are always lower than or equal to $f_{k}^{\gamma }\left(S,t\right)$ and $r_{k}^{\gamma }\left(S^{o},t\right)$ in Table 10, the optimal objective value of the problem with linear work progress constitutes a lower bound for the objective value of the respective problem with stepwise work progress.

### A.3. Mathematical Formulation

Based on the notation and definitions above, we mathematically formulate the RCPSP/c as with Λ depending on the type of objective function:

$$\Lambda =\left\{ \begin{array}{cc} z=S_{I+1} & \text{in}\;\text{the}\;\text{case}\;\text{of}\;\text{project}\;\text{makespan}\;\text{minimization},\\ z^{\prime }=\max\left\{S_{I+1},S_{o_{J+1}}^{o}\right\} & \text{in}\;\text{the}\;\text{case}\;\text{of}\;\text{total}\;\text{makespan}\;\text{minimization}. \end{array}\right.$$

Constraints (1b) are the precedence constraints. Constraints (1c) are the flow-induced precedence constraints. Constraints (1d) are the release constraints. Note that for instances with granular operations, $d_{jj^{\prime }}^{min}=d_{j}$ holds. This is why the formulation of (1c) in [1], Section 5, differs from the formulation here. Constraints (1e) are the renewable resource constraints. Constraints (1f) are the cumulative resource constraints. Constraints (1g), (1h), and (1i) define the domains of the decision variables.
